# Cytofluorometric determination of thymidine kinase activity in a mixture of normal and neoplastic cells.

**DOI:** 10.1038/bjc.1993.187

**Published:** 1993-05

**Authors:** M. Hengstschläger, E. Wawra

**Affiliations:** Vienna Biocenter, Institute for Molecular Biology, Austria.

## Abstract

A cytofluorometric assay allowing the measurement of thymidine phosphorylation in single cells had been established (Hengstschläger & Wawra, 1993). This assay enables us to correlate intracellular thymidine kinase (TK) activity with the DNA content of single cells. Enzyme activity levels from neuroblastoma cells and normal fibroblasts derived from the same patient were determined. Using this cytofluorometric assay in a mixture of both cell types the neoplastic cells could be distinguished from the normal fibroblasts because of their higher TK level. A human lymphoblastoid cell line was compared with the cell line KG-1, derived from an acute myelogenous leukaemia, in the same way. The increased enzyme activity enabled us to detect KG-1 cells in a mixture with an 10,000-fold excess of Epstein Barr virus transformed lymphocytes.


*Br. J. Cancer* (1993), **67**, 1022–1025 © Macmillan Press Ltd., 1993

### Cytofluorometric determination of thymidine kinase activity in a mixture of normal and neoplastic cells

M. Hengstschläger & E. Wawra

*Vienna Biocenter, Institute for Molecular Biology, Dr. Bohrgasse 9, A-1030 Vienna, Austria.*

**Summary** A cytofluorometric assay allowing the measurement of thymidine phosphorylation in single cells had been established (Hengstschläger & Wawra, 1993). This assay enables us to correlate intracellular thymidine kinase (TK) activity with the DNA content of single cells.

Enzyme activity levels from neuroblastoma cells and normal fibroblasts derived from the same patient were determined. Using this cytofluorometric assay in a mixture of both cell types the neoplastic cells could be distinguished from the normal fibroblasts because of their higher TK level. A human lymphoblastoid cell line was compared with the cell line KG-1, derived from an acute myelogenous leukaemia, in the same way. The increased enzyme activity enabled us to detect KG-1 cells in a mixture with an 10,000-fold excess of Epstein Barr virus transformed lymphocytes.

Thymidine kinase, ATP:thymidine 5'phosphotransferase (EC 2.7.1.21), converts thymidine (Thd) to thymidine monophosphate (TMP). This enzyme of the pyrimidine nucleotide salvage pathway occurs mainly in two forms in human tissue, TK1 and TK2 (Shows *et al.*, 1979). The cytosolic enzyme TK1, dominates in replicating cells, but is absent in resting cells (Bello, 1974). Therefore high levels of TK1 activity are found in foetal and neoplastic cells, but its activity is low in non-growing adult tissue (Gordon *et al.*, 1968; Herzfeld & Greengard, 1980; Bardot *et al.*, 1991). The second type of the enzyme, TK2, is of mitochondrial origin and causes the lower and constant level of TK activity in resting cells (Adler & McAuslin, 1974).

A relationship was observed between elevated serum TK levels and cancer stage and prognosis in patients with non-Hodgkin's and Hodgkin's lymphoma (Gronowitz *et al.*, 1983), in patients with acute myelogenous leukaemia (Hagberg *et al.*, 1984), chronic lymphocytic leukaemia (Källander *et al.*, 1984) and multiple myeloma (Simonsson *et al.*, 1985). The increase of serum total TK activity appears to originate from the tumour cells and seems to reflect intracellular TK1 activity (O'Neill *et al.*, 1987; Källander *et al.*, 1987). All these observations suggest that quantitative analyses of TK would distinguish malignant from normal cells.

In order to study the regulation of TK in connection with cellular proliferation, we have established a cytofluorometric assay that allows the measurement of thymidine phosphorylation in single cells and its correlation with the cellular DNA content (Hengstschläger & Wawra, 1993). We synthesised a fluorescent thymidine analogue that is phosphorylated by TK in cell free extracts. This was taken up and phosphorylated by cells in culture. Therefore the cytofluorometric signal of the accumulated fluorochrome reflects the TK activity of the cell (Wawra, 1988). Simultaneous measurement of the cellular DNA content allows the correlation of TK activity with the phase of growth in mixed cell populations and enables the detection of subpopulations with elevated TK activity in an excess of normal cells.

Using the traditional radioactive TK assay we determined the total intracellular TK activity of four cell types: (1) normal human fibroblasts derived from a neuroblastoma patient, (2) neuroblastoma cells from the same patient, (3) a human lymphoblastoid cell line and (4) the human cell line KG-1, derived from an acute myelogenous leukaemia. In mixtures of either neuroblastoma cells and normal fibroblasts or cells of the lymphoblastoid cell line and KG-1 we distinguished neoplastic from normal cells by their intracellular TK activity using this cytofluorometric assay. The fraction of tumour cells in the mixtures was determined.

### Material and methods

### *Chemicals*

5-amino-2-deoxyuridine (AUdR) and 5-dimethylamino-1-naphthalene-sulfonyl-chloride (DANS) were obtained from Sigma, Deisenhofen, West Germany. Ethidium Bromide (EtBr) was purchased from SERVA, Heidelberg, West Germany. Synthesis and purification of the fluorescent thymidine analogue AUdR/DANS are described previously (Hengstschläger & Wawra, 1993).

### *Cells*

Human neuroblastoma cells, derived from a female patient with an aggressive tumour, were cultivated after tumour resection. During cultivation the amount of normal fibroblasts in this heterogeneous population of normal and neoplastic cells increased. The mixture was analysed by the cytofluorometric TK assay after growing *in vitro* for 6 months. We were also able to cultivate neuroblastoma cells and normal fibroblasts of the same patient separately. This allowed the characterisation and analysis of these two cell types.

A normal human lymphocyte cell line was established by transforming phytohemaglutinin stimulated peripheral blood cells with Epstein Barr virus (EBV). The cell line KG-1 (CCL246) was obtained from the American Type Culture Collection, Rockville, Maryland. Tissue cultures were maintained at 37°C, 5%$CO_2$ in 75 $cm^2$ flasks containing RPMI medium supplemented with 10% foetal calf serum. Cells were passaged after reaching a density of about $2-4 \times 10^6$ cell per ml. The cell lines were routinely screened for the absence of mycoplasma.

### *Thymidine kinase assay in cell free extracts*

Cells were lysed in 10 mM Tris-HCl (pH 7.5), 160 mM KCl, 250 mM sucrose, 1.5 mM Mg $Cl_2$, 50 mM ε-aminocapronic acid and 3 mM β-mercaptoethanol containing 0.5% Nonidet-P40 by keeping 10 min at 0°C. Conventional thymidine kinase assays using radioactive thymidine were performed as described previously (Wawra *et al.*, 1981). Protein concentrations in the extracts were determined according to the method of Bradford (1976).

Correspondence: E. Wawra.
Received 8 September 1992; and in revised form 22 December 1992.

*Simultaneous measurement of intracellular TK activity and DNA content*

Detailed information about the cytofluorometric TK assay is described by Hengstschläger and Wawra (1993). Cells were exposed to 1.5 $\mu mol\,l^{-1}$ AUdR/DANS for 1 h in serum free RPMI medium. Thereafter, the cells were trypsinised, centrifuged and the pellet was resuspended in PBS.25 $\mu mol\,l^{-1}$ EtBr was added to stain DNA. Fluorescence intensity reflecting thymidine kinase activity and DNA content were simultaneously measured in a Partec PAS-II flow cytometer.

### Results

Total intracellular TK activity measured in extracts of four cell types are presented in Table I. Whereas no great difference between the enzyme levels of normal fibroblasts and Epstein Barr virus transformed lymphocytes could be detected, these results indicate that TK activity in the analysed neoplastic cells is higher than in the corresponding normal cells. The ratio of enzyme activities in the matched pair of normal fibroblasts and neuroblastoma cells, derived from the same patient, can be compared with the ratio in the pair of the lymphoblastoid cell line and KG-1.

DNA content and TK activity of normal human fibroblasts were measured simultaneously in the Flow Activated Cell Analyser (Figure 1). Starting from a low level in G1, TK activity increases during S-phase and declines in G2 (Figure 1b). Determination of DNA content (Figure 1a) leads to a typical distribution of diploid logarithmically growing cells. Staining with EtBr *in vivo* caused a more diffuse pattern than e.g. DAPI (4,6-diamino-2-phenylindol-dihydrochloride) staining in fixed cells. For this analysis it is not possible to use DAPI to stain DNA, because the emission of DAPI can not be separated from the emission of the fluorescent thymidine analogue. Fixation of the cells before the flow cytometric measurement must also be avoided. After fixation the thymidine analogue would permeate out of the cell and the intracellular fluorescence intensity would not reflect the TK activity anymore (Hengstschläger & Wawra, 1993).

Cytofluorometric analysis of neuroblastoma cells, derived from the same patient, is shown in Figure 2. The tetraploidy of these cells could be confirmed by cytogenetic analysis (data not shown). Although intracellular TK activity varied more than in the compared fibroblasts, the enzyme level in G1, S and G2-phase was consistantly higher in these neuroblastoma cells. The ratio of TK activities in neoplastic to normal cells (T/N = 5.66), determined by conventional radioactive assay, could be verified.

Figure 3 presents the analysis of a mixture of normal fibroblasts and neuroblastoma cells after growing *in vitro* for 6 months. The relation between normal and neoplastic cells is about 1:10. Looking at the DNA distribution (Figure 3a) it is hard to distinguish between these two cell types. The first large DNA peak represents diploid fibroblasts in G1 phase,

**Table I** Comparison of TK activity from (a) matched normal human fibroblasts and neuroblastoma cells and (b) EBV transformed human lymphocytes and a cell line obtained from an acute myelogenous leukaemia

| *Cell type* | *TK activity*[a] | *T/N*[b] |
|---|---|---|
| (a) | | |
| Normal fibroblasts | 191 | 5.7 |
| Neuroblastoma | 1081 | |
| (b) | | |
| EBV transformed lymphocytes | 184 | 4.9 |
| KG-1 | 896 | |

[a]Specific TK activity is given in pmol TMP formed per mg protein per h. [b]Ratio of TK activities in a 'matched' pair of neoplastic (T) and normal (N) cells.

**Figure 1** Simultaneous flow cytometric measurement of DNA content and TK activity in normal human fibroblasts. **a**, Distribution of DNA (abscissa) versus cell number (ordinate). **b**, Content of DNA (abscissa) versus intracellular accumulation of the fluorescent thymidine analogue (= TK activity)(ordinate).

whereas the second peak originates from G1 of the tetraploid tumour cells. The analysis of TK enzyme levels lead to a clear cut between normal and neuroblastoma cells (Figure 3b).

To demonstrate that it is possible to discriminate cells, which have identical DNA content, by their different TK activities, we mixed Epstein Barr virus transformed lymphocytes with cells of the acute myelogeneous leukaemia line KG-1. Different mixtures of these two cell types were analysed. In Figure 4 the ratio of KG-1 cells to lymphocytes is 1:1000. It is not possible to distinguish these two cell lines by their DNA content (Figure 4a). A good separation of normal and leukaemic cells can be achieved by the flow cytometric measurement of TK enzyme levels (Figure 4b).

This assay makes possible the detection of 1 KG-1 cell in 10,000 Epstein Barr virus transformed lymphocytes (Figure 5). The limitating step of these analyses is not the relation between the two cell types but the total amount of cells that can be analysed. About 30 to 50 tumour cells can be detected as a subpopulation with elevated TK activity in an excess of normal cells.

### Discussion

The correlation of increased TK level with cancer stage and prognosis is well documented (Gronowitz *et al.*, 1983; Hagberg *et al.*, 1984; Källander *et al.*, 1984; Simonsson *et al.*, 1985). The value of studying total intracellular TK activity originates in the hypothesis that elevated serum TK level

**Figure 2** Simultaneous flow cytometric measurement of DNA content and TK activity in tetraploid neuroblastoma cells. (See Figure 1 for details).

**Figure 4** Flow cytometric analysis of DNA content and TK activity in a mixture of Epstein Barr virus transformed lymphocytes and the acute myelogenous leukaemia line KG-1. Lymphoblastoid cells and leukaemic cells were mixed 1000 to 1, the measured ratio by flow cytometry was 915:1. (see Figure 1 for details).

reflects the increased cellular enzyme status (O'Neill *et al.*, 1987; Källander *et al.*, 1987). The high intracellular TK activity in neoplastic cells may be caused by (1) a longer S-phase in relation to G1 and G2 phase in their cell cycle, (2) a higher increase of TK in S, starting from a low level in G1 or (3) a consistently higher TK in G1, S and G2-phase. Our simultaneous analysis of DNA content and TK activity of neuroblastoma cells compared to matched normal fibroblasts supports the idea of elevated TK levels in G1, S and G2-phase in cancer cells. Moreover the well known increase of TK activity at the G1/S boundary could be detected in neuroblastoma cells. Gordon *et al.* (1968) published (e.g.) a ratio T/N = 2.53 of enzyme activities in the case of Ewing's sarcoma compared to normal bone tissue. Our results observe that neuroblastoma cells, a comparable solid tumour, display 5.7-fold higher TK level than normal fibroblasts, derived from the same patient. This larger increase can be caused by the tetraploidy of the tumour cells in comparison to the matched diploid fibroblasts. A correlation between the relative numbers of chromosomes and the activities of enzymes encoded by their genes was shown for thymidylate synthetase, thymidine kinase and galactokinase (Bardot *et al.*,

**Figure 3** Flow cytometric analysis of DNA contents and TK activity in a mixture of normal, diploid fibroblasts and tetraploid neuroblastoma cells, derived from the same patient. Normal and tumour cells were mixed 1:10, the measured ratio by flow cytometry was 1:10.4 (see Figure 1 for details).

**Figure 5** Visualisation of a low fraction of neoplastic cells. On the left side the flow cytometric analysis of a large amount of EBV transformed lymphocytes is shown. The same cells were mixed with KG-1 cells in the ratio of 10,000 to 1 (measured ratio by flow cytometry 9368:1). The simultaneous cytometric measurement of DNA content and TK activity of this mixture is shown on the right side. (see Figure 1 for details).

1991). Beside other biological parameters including MYCN (formerly N-*myc*) amplification, chromosome 1 aberrations and ploidy level (Brodeur, 1990), TK activity change could be a useful clinical tool in neuroblastoma. In connection with these tumour markers intracellular TK activity could provide additional information about the proliferation rate of cells. It would be of great interest if there exists a correlation between the prognosis and the level of TK activity in neuroblastoma. In mixtures of a lymphoblastoid cell line and the acute myelogenous leukaemia line KG-1, we were able to detect 1 cell with elevated TK activity in 10,000 normal cells. We chose this artificial mixture to demonstrate that these two lines, which are absolutely identical in the DNA pattern, can be discriminated by their TK activities. Unstimulated human lymphocytes isolated from a healthy donor display an almost undetectable intracellular TK activity level (data not shown). Therefore the TK change between normal nondividing lymphocytes and leukaemic cells is very high. The amount of neoplastic cells should be easier to determine in peripheral blood, isolated from leukaemia patients, than in the used mixture of proliferating lymphocytes and KG-1.

In comparison to the traditional radioactive approach the advantages of the described new cytofluorometric TK assay are obvious. This assay is non radioactive, not time consuming and very sensitive. The simultaneous measurement of the cellular DNA content enables the correlation of TK activity with the phase of growth in any mixed cell population. There is reason to believe, that this method is useful for other applications besides cell cultures, e.g. primary cells, isolates of solid tumours etc. After incubation with the fluorescent thymidine analogue, without DNA staining, the cells are still alive.

In future we plan to use a fluorescent activated sorting system in order to find out if it is possible to separate neoplastic cells from normal cells by their TK activity.

The authors wish to thank Sabine Strehl, CCRI, Vienna for providing the cells of the neuroblastoma patient. Claudia Denk provided excellent technical assistance. This study was supported by the 'Fonds zur Förderung der Wissenschaftlichen Forschung', project number P7795-MED, and by P7770-MED. The cytofluorometer was a donation from the 'Jubiläumsfonds der Österreichischen Nationalbank'.

### References

ADLER, R. & MCAUSLIN, B.R. (1974). Expression of thymidine kinase variants is a function of the replicative state of cells. *Cell*, **2**, 113-117.

BARDOT, V., LUCCIONI, C., LEFRANCOIS, D., MULERIS, M. & DUTRILLAUX, B. (1991). Activity of thymidylate synthetase, thymidine kinase and galactokinase in primary and xenografted human colorectal cancers in relation to their chromosomal patterns. *Int. J. Cancer*, **47**, 670-674.

BELLO, L.J. (1974). Regulation of thymidine kinase synthesis in human cells. *Exp. Cell Res.*, **89**, 263-274.

BRADFORD, M.M. (1976). A rapid and sensitive method for the quantitation of microgram quantities of protein utilizing the principle of protein-dye binding. *Anal. Biochem.*, **72**, 248-254.

BRODEUR, G.M. (1990). Neuroblastoma: clinical significance of genetic abnormalities. *Cancer Surveys*, **9**, 673-688.

GORDON, H.L., BARDOS, T.J., CHMIELEWICZ, Z.F. & AMBRUS, J.L. (1968). Comparative study of the thymidine kinase activity and thymidylate kinase activities and of the feedback inhibition of thymidine kinase in normal and neoplastic tissue. *Cancer Res.*, **28**, 2068-2077.

GRONOWITZ, J.S., HAGBERG, H., KÄLLANDER, C.F.R. & SIMONSSON, B. (1983). The use of serum deoxythymidine kinase as a prognostic marker, and in the monitoring of patients with non-Hodgkin's lymphoma. *Br. J. Cancer*, **47**, 487-495.

HAGBER, G., GRONOWITZ, J.S., KILLANDER, A., KALLANDER, C.F.R., SIMONSSON, B., SUNDSTROM, C. & DBERG, G. (1984). Serum thymidine kinase in acute leukaemia. *Br. J. Cancer*, **49**, 537-540.

HENGSTSCHLÄGER, M. & WAWRA, E. (1993). A cytofluorometric assay for the determination of thymidine uptake and phosphorylation in living cells. *Cytometry*, **14**, 39-45.

HERZFELD, A. & GREENGARD, O. (1980). Enzyme activities in human fetal and neoplastic tissues. *Cancer*, **46**, 2047-2054.

KÄLLANDER, C.F.R., SIMONSSON, B., HAGBERG, H. & GRONOWITZ, J.S. (1984). Serum deoxythymidine kinase gives prognostic information in chronic lymphocytic leukaemia. *Cancer*, **54**, 2450-2455.

KÄLLANDER, C.F.R., SIMONSSON, B., GRONOWITZ, J.S. & NILSSON, K. (1987). Serum deoxythymidine kinase correlates with peripheral lymphocyte thymidine uptake in chronic lymphocytic leukemia. *Eur. J. Haematol.*, **38**, 331-337.

O'NEILL, K.L., ABRAM, W.P., HANNIGAN, B.M. & MCKENNA, P.G. (1987). Elevated serum and mononuclear leukocyte thymidine kinase activities in patient with cancer. *Ir. Med. J.*, **80**, 264-268.

SHOWS, T.B., ALPER, C.A., BOOTSMA, D., DORF, M., DOUGLAS, T., HUISMAN, T., KIT, S., KLINGER, H.P., KOZAK, C., LALLEY, P.A., LINDSLEY, D., MCALPINE, P.J., MCDOUGALL, J.K., MEERA KHAN, P., MEISLER, M., MORTON, N.E., OPITZ, J.M., PARTRIDGE, C.W., PAYNE, R., RODERICK, T.H., RUBINSTEIN, P., RUDDLE, F.H., SHAW, M., SPRANGER, J.W. & WEISS, K. (1979). International system for human gene nomenclature. *Cytogenet. Cell Genet.*, **25**, 96-116.

SIMONSSON, B., KÄLLANDER, C.F.R., BRENNING, G., KILLANDER, A. & GRONOWITZ, J.S. (1985). Evaluation of serum deoxythymidine kinase as a marker in multiple myeloma. *Br. J. Haematol.*, **61**, 215-224.

WAWRA, E. (1988). Microinjection of deoxynucleotides into mouse cells: No evidence that precursor for DNA synthesis are channeled. *J. Biol. Chem.*, **263**, 9908-9912.

WAWRA, E., PÖCKL, E., MÜLLNER, E. & WINTERSBERGER, E. (1981). Effect of sodium butyrate on induction of cellular and viral DNA synthesis in polyoma virus-infected mouse kidney cells. *J. Virol.*, **38**, 973-981.


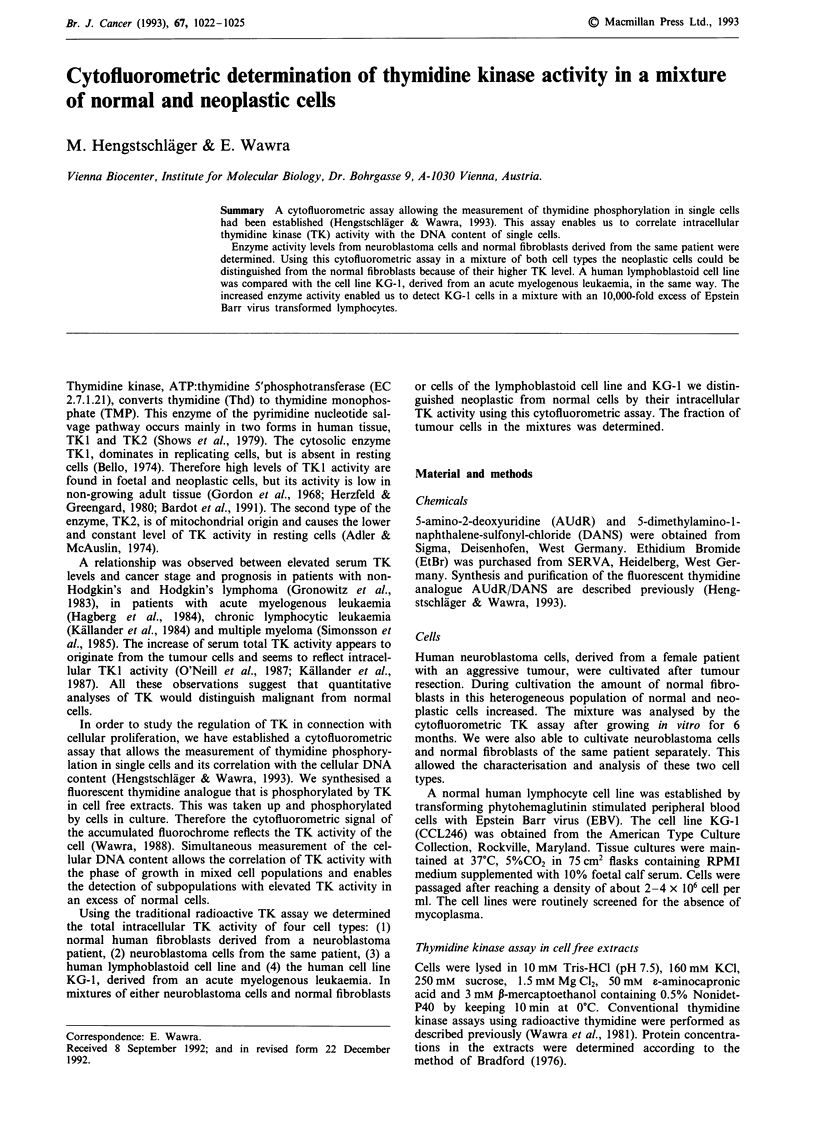

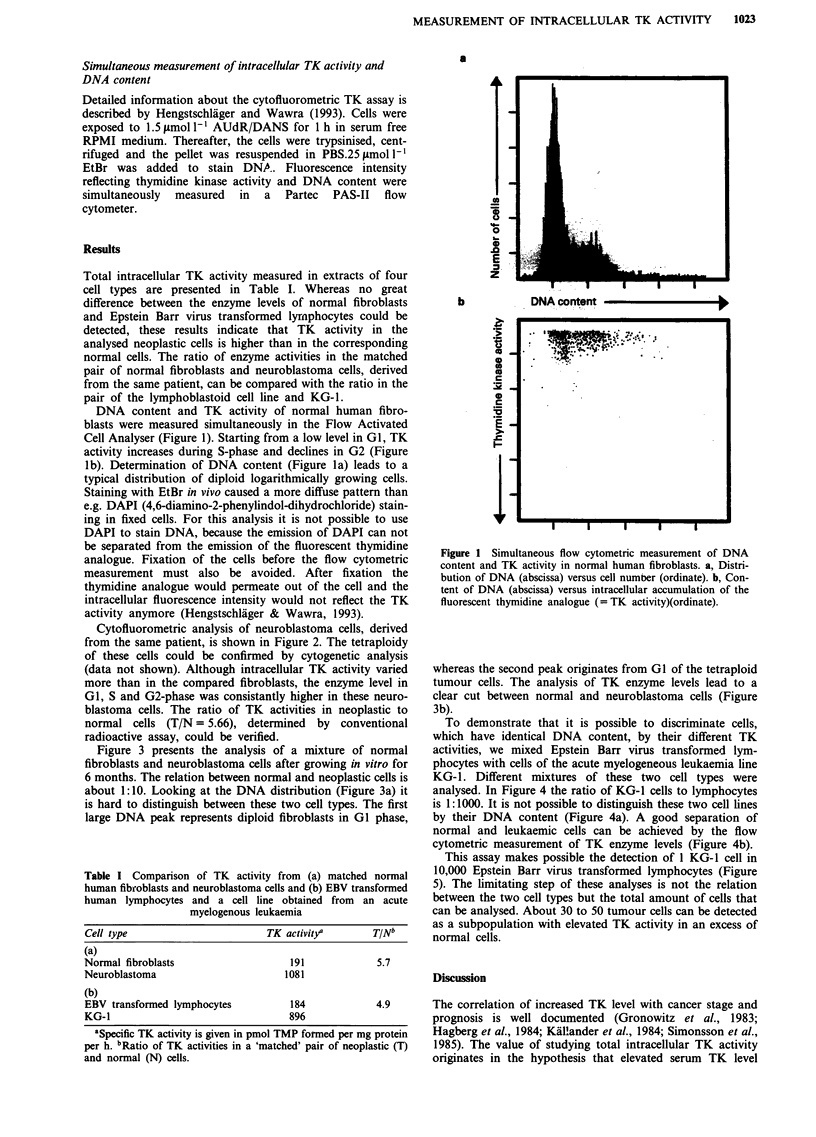

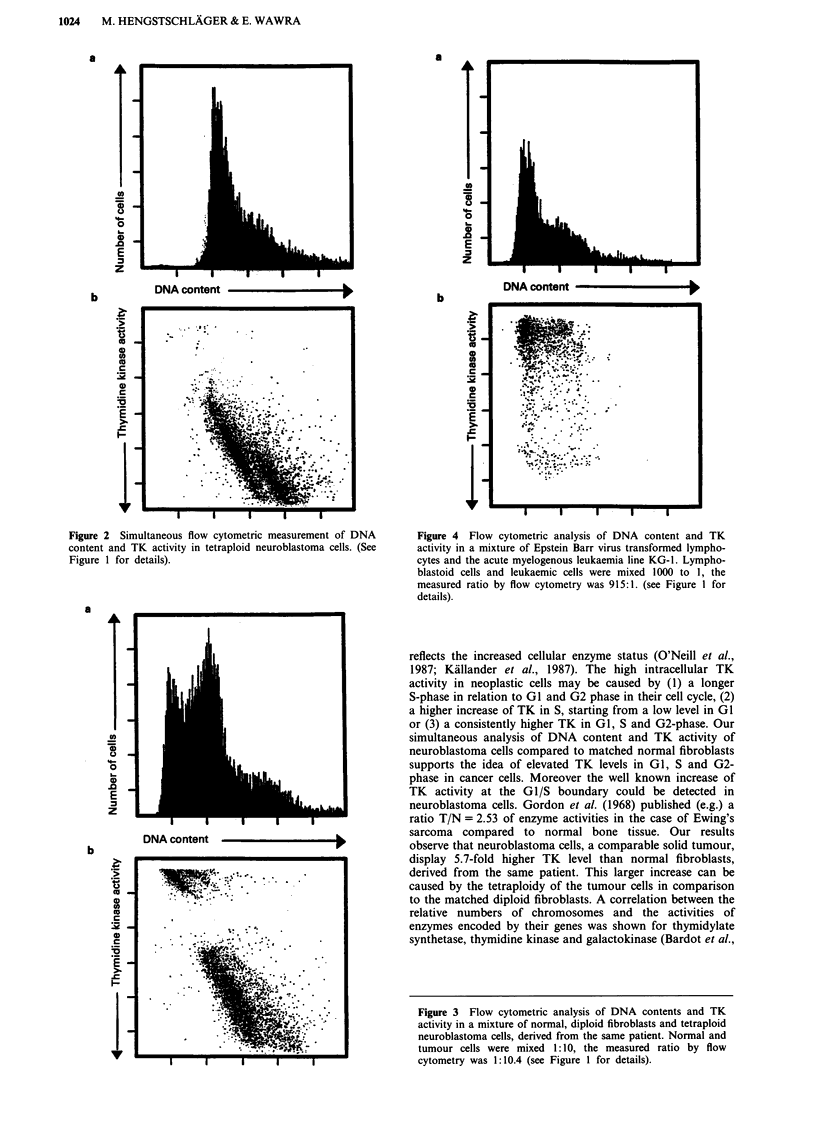

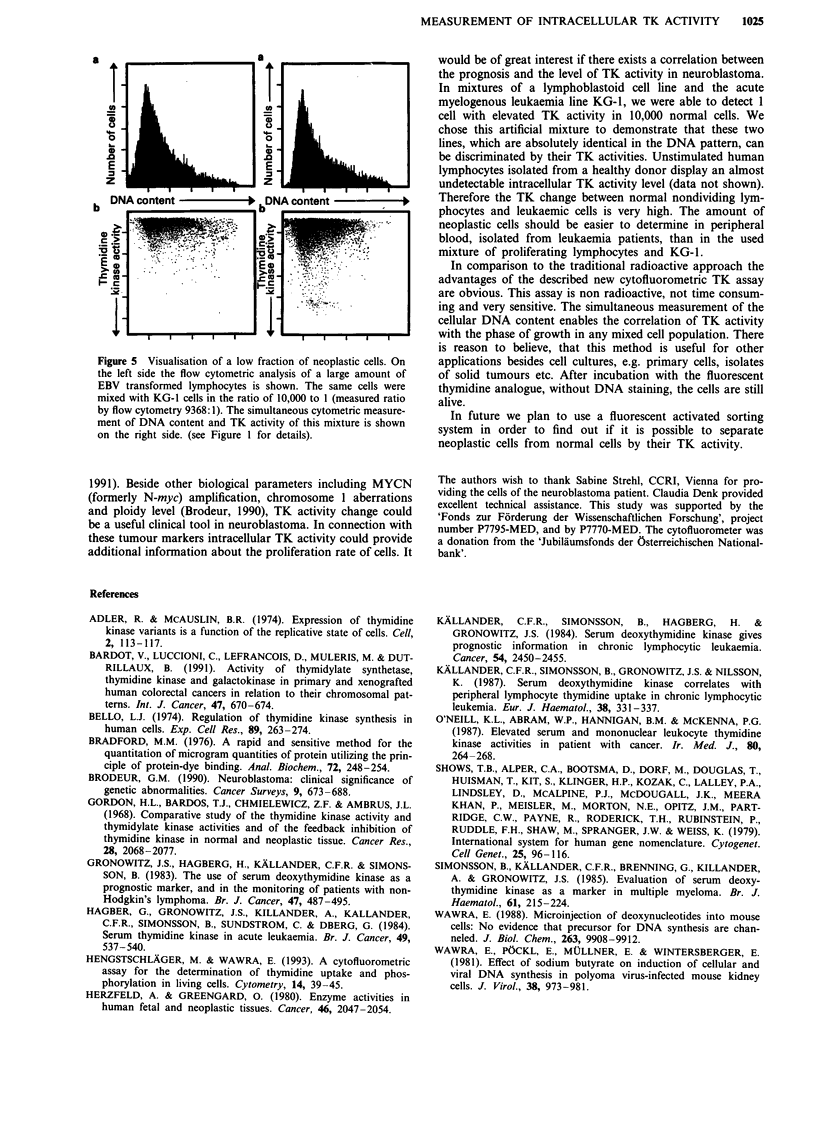


## References

[OCR_00431] Adler R., McAuslan B. R. (1974). Expression of thymidine kinase variants is a function of the replicative state of cells.. Cell.

[OCR_00438] Bardot V., Luccioni C., Lefrançois D., Muleris M., Dutrillaux B. (1991). Activity of thymidylate synthetase, thymidine kinase and galactokinase in primary and xenografted human colorectal cancers in relation to their chromosomal patterns.. Int J Cancer.

[OCR_00443] Bello L. J. (1974). Regulation of thymidine kinase synthesis in human cells.. Exp Cell Res.

[OCR_00447] Bradford M. M. (1976). A rapid and sensitive method for the quantitation of microgram quantities of protein utilizing the principle of protein-dye binding.. Anal Biochem.

[OCR_00452] Brodeur G. M. (1990). Neuroblastoma: clinical significance of genetic abnormalities.. Cancer Surv.

[OCR_00456] Gordon H. L., Bardos T. J., Chmielewicz Z. F., Ambrus J. L. (1968). Comparative study of the thymidine kinase and thymidylate kinase activities and of the feedbach inhibition of thymidine kinase in normal and neoplastic human tissue.. Cancer Res.

[OCR_00465] Gronowitz J. S., Hagberg H., Källander C. F., Simonsson B. (1983). The use of serum deoxythymidine kinase as a prognostic marker, and in the monitoring of patients with non-Hodgkin's lymphoma.. Br J Cancer.

[OCR_00469] Hagberg H., Gronowitz S., Killander A., Källander C., Simonsson B., Sundström C., Oberg G. (1984). Serum thymidine kinase in acute leukaemia.. Br J Cancer.

[OCR_00475] Hengstschläger M., Wawra E. (1993). Cytofluorometric assay for the determination of thymidine uptake and phosphorylation in living cells.. Cytometry.

[OCR_00480] Herzfeld A., Greengard O. (1980). Enzyme activities in human fetal and neoplastic tissues.. Cancer.

[OCR_00490] Källander C. F., Simonsson B., Gronowitz J. S., Nilsson K. (1987). Serum deoxythymidine kinase correlates with peripheral lymphocyte thymidine uptake in chronic lymphocytic leukemia.. Eur J Haematol.

[OCR_00484] Källander C. F., Simonsson B., Hagberg H., Gronowitz J. S. (1984). Serum deoxythymidine kinase gives prognostic information in chronic lymphocytic leukemia.. Cancer.

[OCR_00496] O'Neill K., Abram P., Hannigan B., McKenna G. (1987). Elevated serum and mononuclear leukocyte thymidine kinase activities in patients with cancer.. Ir Med J.

[OCR_00507] Shows T. B., Alper C. A., Bootsma D., Dorf M., Douglas T., Huisman T., Kit S., Klinger H. P., Kozak C., Lalley P. A. (1979). International system for human gene nomenclature (1979) ISGN (1979).. Cytogenet Cell Genet.

[OCR_00512] Simonsson B., Källander C. F., Brenning G., Killander A., Ahre A., Gronowitz J. S. (1985). Evaluation of serum deoxythymidine kinase as a marker in multiple myeloma.. Br J Haematol.

[OCR_00518] Wawra E. (1988). Microinjection of deoxynucleotides into mouse cells. No evidence that precursors for DNA synthesis are channeled.. J Biol Chem.

[OCR_00523] Wawra E., Pöckl E., Müllner E., Wintersberger E. (1981). Effect of sodium butyrate on induction of cellular and viral DNA syntheses in polyoma virus-infected mouse kidney cells.. J Virol.

